# Distribution of Virulence Markers among *Vibrio vulnificus* Isolates of Clinical and Environmental Origin and Regional Characteristics in Japan

**DOI:** 10.1371/journal.pone.0055219

**Published:** 2013-01-30

**Authors:** Nana Yokochi, Shigemitsu Tanaka, Kouichi Matsumoto, Hirotaka Oishi, Yukihiro Tashiro, Yu Yoshikane, Mikio Nakashima, Kohzo Kanda, Genta Kobayashi

**Affiliations:** 1 Department of Applied Biochemistry and Food Science, Faculty of Agriculture, Saga University, Saga, Japan; 2 Biomaterials and Commodity Chemicals Research Division, Osaka Municipal Technical Research Institute, Osaka, Japan; 3 Department of Anesthesiology and Critical Care Medicine, Faculty of Medicine, Saga University, Saga, Japan; 4 Institute of Advanced Study, Kyusyu University, Higashi-ku, Fukuoka, Japan; 5 Tosa Food Business Creator Project Team, Kochi University, Nankoku, Kochi, Japan; The Australian National University, Australia

## Abstract

**Background:**

*Vibrio vulnificus* is an opportunistic human pathogen that is widely distributed in estuarine environments and is capable of causing necrotizing fasciitis and sepsis. In Japan, based on epidemiological research, the incidences of *V. vulnificus* were concentrated in Kyusyu, mainly in coastal areas of the Ariake Sea. To examine the virulence potential, various genotyping methods have recently been developed. This study aimed to investigate the distribution of virulence markers among *V. vulnificus* isolates of clinical and environmental origin in three coastal areas with different infection incidences and to determine whether these isolates have the siderophore encoding gene *viuB*.

**Methodology/Principal Findings:**

We examined the distribution of genotypes of the 16S ribosomal ribonucleic acid (rRNA) gene, *vvhA*, *vcg*, and capsular polysaccharide (CPS), and the presence of *viuB* in 156 isolates collected from patients and environmental samples in Japan. The environmental samples were collected from three coastal areas: the Ariake Sea, Ise & Mikawa Bay, and Karatsu Bay. The results showed disparity in the ratios of genotypes depending on the sample origins. *V. vulnificus* isolates obtained from patients were classified into the clinical type for all genotypes. In the environmental isolates, the ratios of the clinical type for genotypes of the 16S rRNA gene, *vvhA*, and *vcg* were in the order of the Ariake Sea>Ise & Mikawa Bay>Karatsu Bay. Meanwhile, CPS analysis showed no significant difference. Most isolates possessed *viuB*.

**Conclusions:**

Many *V. vulnificus* belonging to the clinical type existed in the Ariake Sea. Three coastal areas with different infection incidences showed distinct ratios of genotypes. This may indicate that the distribution of clinical isolates correlates with the incidence of *V. vulnificus* infection.

## Introduction

*Vibrio vulnificus* is a gram-negative halophilic bacterium, which inhabits warm coastal and estuarine waters worldwide. This microorganism infects humans by the consumption of contaminated seafood or contact of a wound with seawater and causes necrotizing fasciitis and sepsis. The mortality rate is high (>50%), and the latency period is short (within 24 to 48 h) [1], [2]. Patients with underlying disease such as liver dysfunction, alcoholic cirrhosis, or hemochromatosis are particularly susceptible to this life-threatening infection [1].

In addition to the United States, where *V. vulnificus* infections occur frequently, *V. vulnificus* infections have been reported in Japan, South Korea, Taiwan, Australia, Israel, and India [3]–[10]. In recent years, infections have been reported in high-latitude areas of the Baltic Sea coast such as Denmark and Sweden, indicating the spread of the organism [11], [12]. In Japan, a recent epidemiological survey showed that 185 cases of *V. vulnificus* infections occurred from 1975 to 2005 [13]. In particular, there were many clinical reports on the western part of Japan. Northern Kyushu (Saga, Nagasaki, Fukuoka, and Kumamoto prefectures), which encloses the Ariake Sea, the inland sea with large rivers, accounted for approximately 40% of all the reported cases (77 cases), whereas Aichi Prefecture, which is located roughly in the center of Japan and encloses the Ise & Mikawa Bay, accounted for about 10% (19 cases). In contrast, there was no report of infection in Karatsu Bay, the northern area of Saga Prefecture in northern Kyushu that encloses the open sea, during our investigation. There have been many reports about environmental factors that influence *V. vulnificus*, e.g., temperature and salinity of seawater in various areas [10], [14]–[16]. The optimal growth environment of *V. vulnificus* is a seawater temperature of ≥20°C and salinity of 15–20‰ [17]. However, a direct correlation of the incidence of *V. vulnificus* infections and other environmental factors (such as pH, dissolved oxygen, turbidity, and chlorophyll a) has not been proven.

*V. vulnificus* is a heterogeneous bacterial species that exhibits variation in strain virulence. In terms of virulence, it has been classified based on phenotypical and serological characteristics [18]–[20]. Recently, because of the need for rapid differentiation of strains with human virulence potential, genotyping systems based on deoxyribonucleic acid (DNA) polymorphisms at some loci have been developed. Nilsson et al. [21] showed that polymorphic variants generally included two genotypes such as the 16S ribosomal ribonucleic acid (rRNA) gene types A and B that significantly correlated with the non-clinical (environmental) and clinical isolates, respectively. Senoh et al. [22] found that the hemolysin gene (*vvhA*) could differentiate between two genotypes, termed type 1 and type 2, correlating with clinical and non-clinical types, respectively. Similarly, random amplification of polymorphic DNA typing by Rosche et al. [23] showed the E-type and C-type sequence variants at the virulence-correlated gene (*vcg*) locus, correlating with the non-clinical and clinical isolates, respectively. Capsular polysaccharide (CPS) is required for virulence, and many reports of lethality in animal models are clearly related to CPS expression [24]–[26]. Chatzidaki-Livanis et al. [27] observed heterogeneity within the CPS operon sequences at the same locus in different strains and referred to these as allele 1 and allele 2. These alleles diverged from sequences encoding hypothetical proteins (HPs). HP1 and HP2 alleles correlated with clinical and non-clinical types, respectively. In addition, multiplex polymerase chain reaction (PCR) studies by Panicker et al. [28] showed that clinical isolates were more likely to be positive for the siderophore-encoding gene *viuB* than environmental isolates. Regarding the prevalence of *viuB*, Bogard et al. [29] found that serum survivability of the *viuB-*positive isolate was greater than that of the *viuB*-lacking ones. Bacterial genotyping methods focusing on differences between clinical and non-clinical types have been performed for individual or two to four combinations of genes; however, genotypic analysis of a combination of five genes participating in *V. vulnificus* infection has not been reported. In addition, genotypic analysis of *V. vulnificus* in areas with different infection incidences based on epidemiological investigation has rarely been performed.

In the present study, we examined the distribution of genotypes of the 16S rRNA gene, *vvhA*, *vcg*, CPS, and the presence of *viuB* among *V. vulnificus* isolates of clinical (16 isolates) and environmental (140 isolates) origin. Furthermore, to determine the genotypes of *V. vulnificus* from areas with different infection incidences, we characterized the isolates collected from the Ariake Sea, Ise & Mikawa Bay, and Karatsu Bay.

## Results

### Genotypic Analysis of *V. vulnificus*

Tables S1, S2 summarize the genetic profiles of all 156 isolates. The 16 clinical isolates are shown in Table S1, and 140 environmental isolates are shown in Table S2. Genotypes of *V. vulnificus* were designated on the basis of 16S rRNA, *vvhA*, *vcg*, and CPS allele types, and the presence or absence of *viuB*. Genotypes of 16S rRNA, *vvhA*, and *vcg* types could be divided into two groups. Genotype of CPS could be divided into two groups; however, approximately 30% of the isolates could not be categorized into either group because PCR amplicon was not detected.

### Genotypic Characterization of Isolates Collected from Patients and the Ariake Sea

The distribution of genotypes and the presence of *viuB* in *V. vulnificus* from the clinical and Ariake Sea isolates are shown in Table 1. Based on 16S rRNA genetic analysis, 15 among the 16 (93.8%) *V. vulnificus* isolates from patients (clinical isolates) belonged to type B. Similarly, in the clinical isolates, both the C-type and allele 1 were classified as the clinical type in terms of the *vcg* type and CPS allele, and the proportion was high (93.8%, 15/16 isolates). All the isolates possessed *viu*B. These results were in agreement with previous reports on the clinical type [21], [23], [29]. The prevalence rate of *vvhA*, which is classified into type 1 (clinical type), was 68.8% (11/16 isolates). Most of the Ariake Sea isolates were classified as type B, type 1, and C-type, which are clinical types in genotype of the 16S rRNA gene, *vvhA* type, and *vcg* type, respectively, and their prevalence rates were 90.5% (105/116 isolates), 85.3% (99/116 isolates), and 91.4% (106/116 isolates), respectively. All the isolates possessed *viuB*. In contrast, the prevalence rate of allele 1, which is the clinical type by CPS analysis, was 50.9%. Thus, the clinical type (the 16S rRNA gene type B, *vvhA* type 1, and *vcg* C-type) was more frequent in genotypes of the Ariake Sea isolates, except by CPS analysis, and its ratios between clinical and non-clinical types showed no statistically significant difference between the Ariake Sea and clinical isolates in all the loci (Table 1). When we subcategorized the isolates from the Ariake Sea as having seafood and non-seafood sources and compared the genotypes, there were no significant differences among both the sources.

**Table 1:** Distribution of genotypes and *viuB* proportion of *V. vulnificus* according to clinical and Ariake Sea isolates.

	% with genotype											% with profile					*viu*Bproportion(%)
	rRNA gene		*vvhA*			*vcg*			CPS			Profile					
	B	A	type 1	type 2	NA^a^	C-type	E-type	C/E	allele 1	allele 2	NA^a^	1	2	4	Untypeable	no profile	
Clinical (n = 16)	93.8	6.3	68.8	31.3	0.0	93.8	6.3	0.0	93.8	6.3	0.0	68.8	6.3	25.0	0.0	0.0	100.0
Ariake Sea (n = 116)	90.5	0.9	85.3	13.8	0.9	91.4	6.9	1.7	50.9	19.0	30.2	81.9	5.2	6.9	2.6	3.4	100.0
Seafood (n = 14)	85.7	14.3	85.7	14.3	0.0	85.7	14.3	0.0	64.3	28.6	7.1	71.4	7.1	7.1	14.3	0.0	100.0
Non-seafood (n = 102)	91.2	8.8	85.3	13.7	1.0	92.2	5.9	2.0	49.0	17.6	33.3	83.3	4.9	6.9	1.0	3.9	100.0

a NA, not amplified.

rRNA, ribosomal ribonucleic acid; CPS, capsular polysaccharide.

### Genotypic Characterization of Three Areas with Different *V. vulnificus* Incidences

**Figure 1:**
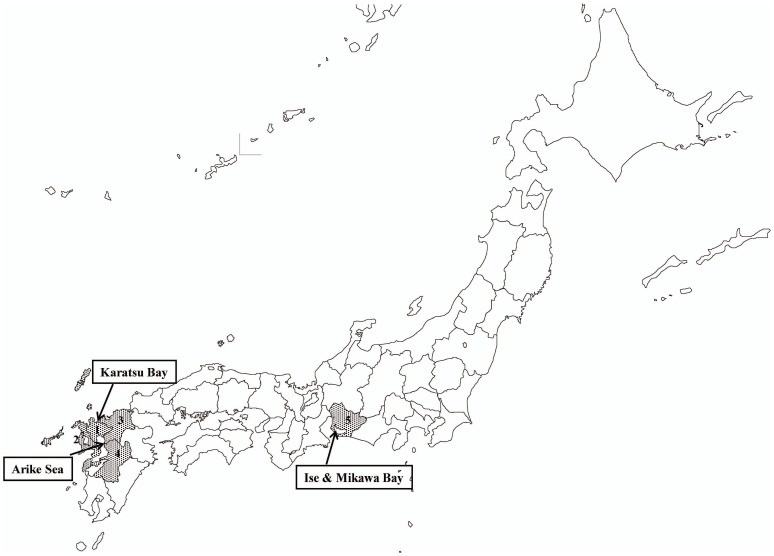
Map of sampling points. The prefectures indicated by dots are areas where *V. vulnificus* infections occurred. 1, Saga; 2, Nagasaki; 3, Fukuoka; 4, Kumamoto; and 5, Aichi prefectures.

The distribution of genotypes and *viuB* prevalence among the environmental *V. vulnificus* isolates from three areas (the Ariake Sea, Ise & Mikawa Bay, and Karatsu Bay; shown in Fig. 1) are shown in Table 2. In the Ariake Sea, Ise & Mikawa Bay, and Karatsu Bay isolates, the prevalence rate of 16S rRNA gene type B (clinical type) was 90.5%, 63.6%, and 38.5%, respectively; that of *vvhA* type 1 (clinical type) was 85.3%, 72.7%, and 38.5%, respectively; and that of *vcg* C-type (clinical type) was 91.4%, 72.7%, and 30.8%, respectively. Two isolates (A-56 and A-68) collected from the Ariake Sea possessed both *vcg* C and *vcg* E. The prevalence rate of *viuB* in the Ariake Sea and Ise & Mikawa Bay isolates was 100%, whereas that in the Karatsu Bay isolates was 84.6%. Two *viuB*-negative isolates (K-5 and K-10) that originated from Karatsu Bay were the non-clinical type in genotypes of 16S rRNA gene, *vvhA*, and *vcg*. In contrast, CPS loci analysis showed no statistically significant difference among areas. The prevalence rate of CPS allele 1 accounted for approximately 50% and 30% of isolates that could not be classified in any area. Except for CPS, geographical disparity was observed in the ratios of genotypes, and clinical types were observed in the order of the Ariake Sea>Ise & Mikawa Bay>Karatsu Bay isolates. Significant differences were found in all loci in the distributions of genotypes in these three areas (*P*<0.01). These results suggested that the prevalence ratios of clinical- and non-clinical-type *V. vulnificus* differed between the three areas in Japan.

**Table 2:** Distribution of genotypes and *viuB* proportion of *V. vulnificus* according to environmental isolate origins.

	% with genotype											% with profile					*viu*Bproportion(%)
	rRNA gene		*vvhA*			*vcg*			CPS			Profile					
	B	A	type 1	type 2	NA^a^	C-type	E-type	C/E	allele 1	allele 2	NA^a^	1	2	4	Untypeable	no profile	
Ariake Sea (n = 116)	90.5	0.9	85.3	13.8	0.9	91.4	6.9	1.7	50.9	19.0	30.2	81.9	5.2	6.9	2.6	3.4	100.0
Seafood (n = 14)	85.7	14.3	85.7	14.3	0.0	85.7	14.3	0.0	64.3	28.6	7.1	71.4	7.1	7.1	14.3	0.0	100.0
Non-seafood (n = 102)	91.2	8.8	85.3	13.7	1.0	92.2	5.9	2.0	49.0	17.6	33.3	83.3	4.9	6.9	1.0	3.9	100.0
Ise & Mikawa Bay	63.6	36.4	72.7	27.3	0.0	72.7	27.3	0.0	63.6	9.1	27.3	63.6	27.3	0.0	9.1	0.0	100.0
Non-seafood (n = 11)																	
Karatsu Bay (n = 13)	38.5	61.5	38.5	61.5	0.0	30.8	69.2	0.0	53.8	15.4	30.8	30.8	61.5	0.0	7.7	0.0	84.6
Seafood (n = 4)	75.0	25.0	75.0	25.0	0.0	50.0	50.0	0.0	50.0	50.0	0.0	50.0	25.0	0.0	25.0	0.0	100.0
Non-seafood (n = 9)	22.2	77.8	22.2	77.8	0.0	22.2	77.8	0.0	55.6	0.0	44.4	22.2	77.8	0.0	0.0	0.0	77.8

a NA, not amplified.

rRNA, ribosomal ribonucleic acid; CPS, capsular polysaccharide.

### Genotypic Profiles According to Areas

**Figure 2:**
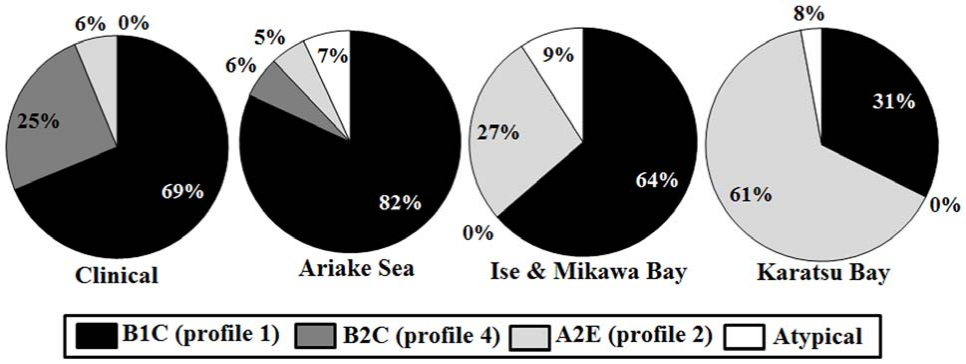
Ratio of genotypic profile according to isolate origins. The ratio of profile of clinical and environmental isolates is shown. The three main genotypic profiles were combined. Profile 1 (clinical type) consisted of 16S rRNA gene type B, *vvhA* type 1, and *vcg* C-type; profile 2 (non-clinical type) consisted of type A, type 2, and E-type; and profile 4 consisted of type B, type 2 and C-type. Other combination types were set to untypeable.

Combinations of the three main genotypic profiles, as described Sanjuán et al. [30], were examined according to the region of isolation. Profile 1 (clinical type) consisted of genotypes of 16S rRNA gene type B, *vvhA* type 1, and *vcg* C-type, whereas profile 2 (non-clinical type) consisted of genotypes of 16S rRNA gene type A, *vvhA* type 2, and *vcg* E-type. The combination of 16S rRNA gene type B, *vvhA* type 2, and *vcg* C-type observed in this study was designated as profile 4 (Profile 3 has already been defined with a different gene profile, i.e., the combination of 16S rRNA gene type AB, *vvhA* type 1, and *vcg* E-type, by Sanjuán et al. [30]). Other combinations were assigned untypeable. The prevalence rate of profiles for the clinical and environmental isolates is shown in Fig. 2. Among the clinical isolates, the prevalence rate of profile 1 was 69%. In the Ariake Sea, Ise & Mikawa Bay, and Karatsu Bay isolates, the percentages of profile 1 were 82%, 64%, and 32%, respectively. In contrast, the prevalence rate of the isolates classified as profile 2 was 6%, 5%, 27%, and 65%, respectively. Profile 4 was only observed in the clinical (25%) and Ariake Sea (6%) isolates. These results also showed that the prevalence rate of clinical-type *V. vulnificus* inhabiting the Ariake Sea was higher than that of the clinical isolates. Among the isolates from the three areas, the genotypic profile of the Karatsu Bay isolates was significantly differed from that of the clinical ones (*P*<0.05). When we subcategorized the environmental isolates from the Ariake Sea and Karatsu Bay as having seafood and non-seafood sources and compared the genotypes and profiles (Table 2), there was no statistically significant difference among both the sources. This suggests that regardless of the different sources such as seafood and non-seafood sources, their genotypes and profiles were present at the same rate.

## Discussion

Although it is clear that *V. vulnificus* inhabits coastal marine waters worldwide, the number of people infected with *V. vulnificus* is less compared with the microbial load. The use of genotyping analysis has been prompted to determine the virulence potential between the clinical-type and non-clinical-type *V. vulnificus* isolates [18]–[20], [31].

We first investigated 16 clinical isolates reported in areas surrounding the Ariake Sea using five biomarkers: the 16S rRNA gene, *vvhA*, *vcg*, CPS, and *viuB*. Many *V. vulnificus* isolates obtained from patients were classified into the clinical type for all genotypes. These distributions were mostly in agreement with the typing results reported previously [21]–[23], [30], suggesting that clinical isolates have essentially the same genotypes in any region.

Interestingly, when genotypes were investigated in terms of *V. vulnificus* isolated from three areas (the Ariake Sea, Ise & Mikawa Bay, and Karatsu Bay) the prevalence rates of clinical and non-clinical types differed in each area (Table 2). The prevalence rate of clinical-type *V. vulnificus* isolated from the Ariake Sea was overwhelmingly high (approximately 90%) and of that isolated from Ise & Mikawa Bay was comparatively high (approximately 70%). Meanwhile, most (approximately 65%) of the isolates from Karatsu Bay belonged to the non-clinical type. Kim and Jeong [32] reported that in seawater, oysters, and sediment of the Hadong area on the southern coast of South Korea, 35% of isolates were classified as A type (non-clinical type) and 65% as B type (clinical type) by 16S rRNA gene analysis. Warner and Oliver [16] reported that in seawater samples from Alligator Bay on the eastern coast of North Carolina, 46.9% of isolates belonged to the non-clinical type (*vcg* E-type) and 53.1% belonged to the clinical type (*vcg* C-type), whereas in Cedar Key, FL, 37.3% of isolates belonged to the non-clinical type and 62.7% belonged to the clinical type. Compared with these results, the number of *V. vulnificus* isolates belonging to the clinical type was much higher in the Ariake Sea. Although the number of *V. vulnificus* isolates obtained from Ise & Mikawa Bay and Karatsu Bay was limited, the distribution of three genotypes (16S rRNA gene, *vvhA*, and *vcg*) was in agreement with each area. Therefore, we concluded that genotypes of *V. vulnificus* differed by region in Japan.

Based on the 16S rRNA gene, *vvhA*, and *vcg*, we classified the isolates into three profiles (Fig. 2). In the clinical isolates, profile 3 (16S rRNA gene type B, *vvhA* type 2, and *vcg* C-type) was observed (25%). In the environmental isolates, profile 4 was observed only in the Ariake Sea (6%) isolates and not in the Ise & Mikawa Bay and Karatsu Bay ones. To our knowledge, there are few reports about profile 4. It can be said that profile 4 was the feature of the clinical isolates reported in areas surrounding the Ariake Sea near Saga Prefecture and the isolates collected from the Ariake Sea in Japan.

On investigating the epidemiological and clinical characteristics of *V. vulnificus* infection reported in Japan, we found that approximately 40% of cases occurred in four prefectures around the Ariake Sea and approximately 10% were reported in Aichi Prefecture, which encloses Ise & Mikawa Bay [14]. In our study, *V. vulnificus* was isolated from the Ariake Sea and Ise & Mikawa Bay, which are semi-closed seas with relatively low salinity and warm seawater temperatures. In contrast, there was no report of *V. vulnificus* infection in the northern area of Saga Prefecture, which encloses Karatsu Bay, during our investigation. Karatsu Bay faces the Sea of Japan, and neither salinity nor seawater temperature seem to be affected by changes in the weather. Therefore, we supposed that the Ariake Sea and Ise & Mikawa Bay were more suitable for *V. vulnificus* than Karatsu Bay. Yahiro et al. reported [33] that more *V. vulnificus* isolates were detected around the inland sea area with many rivers (where *V. vulnificus* infections were reported) than in the open sea area in Kumamoto Prefecture. Thus, inhabitation of a large number of *V. vulnificus* may be responsible for the large number of infections reported. In addition to this report, we found that statistically more clinical-type *V. vulnificus* existed in the epidemic areas of infection such as the Ariake Sea and Ise & Mikawa Bay, whereas more non-clinical-type *V. vulnificus* existed in the non-epidemic area of infection such as the Karatsu Bay as described above. Therefore, our results suggest that genotype distributions of *V. vulnificus* in the region are related to infection incidence.

In CPS analysis, a difference was not seen in the distribution of two alleles among three areas; 50%–60% of the isolates belonged to the clinical type (allele 1) and 10%–20% belonged to the non-clinical type (allele 2). Interestingly, in approximately 30% of the isolates, both alleles 1 and 2 could not be identified by PCR in all three areas. Han et al. reported that approximately 20% of the isolates could not be amplified by the same method [34]. Chatzidaki-Livanis et al. reported that one environmental strain could not be divided either allele 1 or 2 in 33 clinical and 35 environmental strains [27]. HP segments including CPS alleles were inserted between *wza* and *wzb* in *V. vulnificus* [27]. Although we tried to confirm an intact HP gene using PCR primers (forward primer: *wza* region, reverse primer: *wzb* region), most isolates could not be amplified (data not shown); therefore, it was proposed that these isolates may not contain the HP gene. Considering that all the isolates collected from patients could be distributed into either allele 1 or allele 2, the existence of this gene may be associated with human infection.

In the present study, we investigated genotypes of *V. vulnificus* isolated from three areas: the Ariake Sea, Ise & Mikawa Bay, and Karatsu Bay. We found that there was regional difference in genotypes of *V. vulnificus* that was relevant to infection rate in each area. In addition, we found that significantly larger numbers of clinical-type *V. vulnificus* existed in the epidemic areas of infection such as the Ariake Sea and Ise & Mikawa Bay, suggesting that the genotype distribution of *V. vulnificus* is related to infection incidence. To date, there are no unique virulent biomarkers because all clinical and environmental isolates are equally virulent in animal and cell culture pathogenesis models [35]. Continuous sampling and multiple analyses may lead to discovery of an effective new virulence biomarker for *V. vulnificus*.

## Materials and Methods

### Bacterial Strains and Culture Preparation

A total of 156 *Vibrio vulnificus* isolates, including 16 clinical and 140 environmental isolates (116 isolated from the Ariake Sea, 11 from Ise & Mikawa Bay, and 13 from Karatsu Bay), were used in this study (Fig. 1). The clinical isolates were collected from patients with *V. vulnificus* infection at hospitals in Saga Prefecture and 19 other emergency hospitals in the region where we have established an information network surrounding the Ariake Sea near Saga Prefecture for *V. vulnificus* infection during the period of 1984–2010. Permission to examine medical records for identifying cases of *V. vulnificus* infection was granted from each institution. Patients were suffering from liver dysfunction (n = 15), and multiple endocrine neoplasia (n = 1), and the mortality rate was 75%. The source of infection was the ingestion of raw fish/shellfish and garnish contacted with raw fish (n = 14) or unknown (n = 2).

Microbial identification of *V. vulnificus* was determined in agar plate cultures and confirmed by biochemical methods from clinical specimens, including blood, blister fluids, and necrotizing tissue samples, at each hospital. The environmental isolates were collected from seawater, mud, oysters, and fish collected from the Ariake Sea and Karatsu Bay in Saga Prefecture and Ise & Mikawa Bay in Aichi Prefecture from April to September in 2001, 2002, and 2007–2011. In particular, a number of samples were collected during the summer months of July–September. No specific permits were required for sampling. The sampling location is not privately-owned or protected in any way, and the field studies did not involve endangered or protected species. The *V. vulnificus* isolates from the Ariake Sea were collected near Saga Prefecture. Oysters were collected, their external surfaces were washed and opened, and the entire oyster tissue was ground with seawater. Seawater (10 ml) was centrifuged, and 9 ml was then discarded. Aliquot (1 ml) was used as sea water sample. Mud was added directly. All the samples were aerobically grown at 30°C or 37°C in modified Zobell broth containing 0.5% peptone and 0.1% yeast extract in seawater (24.3 PSU). The cultivate solution was then plated onto the ES Vibrio agar plate (Eiken Chemical Co., Ltd., Tokyo, Japan) and incubated at 30°C for 15–24 h. Perspective *V. vulnificus* colonies on plates were picked onto a new plate. All the isolates were aerobically grown at 30°C or 37°C in modified Zobell broth. Genomic DNA was isolated from each culture using a DNA isolation kit (QIAamp® DNA Mini Kit; Qiagen, Hilden, Germany) according to the manufacturer’s instructions and used as template in PCR assays.

### Genotyping

The V1–V3 region of the bacterial 16S rRNA gene was amplified from genomic DNA isolated from each strain using PCR with a pair of eubacterial universal primers: 8UA (5′-AGAGTTTGATCCTGGCTCAG-3′) and 519B (5′-ATTACCGCSGCTGCTG-3′) [36], [37]. PCR was performed as described by Nilsson et al. [21]. Genotypes were determined depending on sequence homology to *V. vulnificus* ATCC27562^T^ representative of type A (GenBank accession number X76333) or *V. vulnificus* C7184 representative of type B (GenBank accession number X76334) as reported previously [21]. An 813-bp segment of the hemolysin gene (*vvhA*) was targeted for PCR amplification using two primer sets of *vvhA*-1F (5′-AGATTAAGTGTGTGTTGCACAAGCGGTG-3′) and *vvhA*-1R (5′-ACCGAAAACAGCGCTGAAGGAAGAACGGTA-3′) pair, and *vvhA*-2F (5′-AAATTAAGTGCGTGCTACACACAAGTGGTG-3′) and *vvhA*-2R (5′-A CTGAGAAGAGTGCTGAAGGGATTACCGTA-3′) pair [22]. *vvhA* amplified with *vvhA*-1F and *vvhA*-1R was designated as type 1 and that amplified with *vvhA*-2F and *vvhA*-2R was designated as type 2. PCR was performed with Ex *Taq* polymerase (Takara Bio, Shiga, Japan) as described previously [22]. Genotyping of *vcg* was performed using PCR according to Rosche et al. [23]. The primers P1 (5′-AGCTGCCGATAGCGATCT-3′) and P3 (5′-CGCTTAGGATGATCGGTG -3′) were used for identification of the C-type isolates. The primers P2 (5′-CTCAATTGACAATGATCT-3′) and P3 were used for identification of the E-type isolates. Genomic DNA from each isolate was subjected to a separate PCR reaction with each of the two primer sets. PCR was performed as described by Rosche et al. [23]. To detect specific CPS alleles, the *V. vulnificus* isolates were examined using PCR according to Han et al. [34]. Two primer pairs were used: primers HP1F (5′-TTTGGGTTTGAAAGGCTTG-3′) and HP1R (5′-GTGCCTTTGCGAAT TTTGAT-3′) were used to detect HP1 in *V. vulnificus* MO6-24/O (CPS allele 1) and primers HP2F (5′-TTCCATCAAACATCGCAGAA-3′) and HP2R (5′-CTTTTGTCCGGCTTCTATGC-3′) were used to detect HP2 in *V. vulnificus* YJ016/O (CPS allele 2) at the same locus. PCR was performed under the conditions described by Han et al. [34]. A 342-bp product was detected in a PCR reaction with the HP1 primer set, and a 152-bp product was detected with the HP2 primer set. PCR detection of *viuB* was performed using a primer set reported by Jones et al. [38]: forward primer (5′-GGTTGGGCACTAAAGGCAGAT-3′) and reverse primer (5′-TCGCTTTCTCCGGGGCGG-3′). PCR was performed with Ex *Taq* polymerase (Takara Bio) as described by Jones et al. [38].

### Statistical Analysis

Statistical analyses were performed using the chi-squared test or Fisher’s exact test. Statistical significance was determined using a *P*-value of 0.05. All analyses were performed using Microsoft Excel (Excel Toukei 2010; Social Survey Research Information, Tokyo, Japan).

## Supporting Information

Table S1 **Clinical isolates in this study along with their respective origins and properties.**
(DOC)

Table S2 **Environmental isolates used in this study along with their respective sources and properties.**
(DOC)
