# Supplementary material for: Distribution of Virulence Markers among Vibrio vulnificus Isolates of Clinical and Environmental Origin and Regional Characteristics in Japan
Source: PLoS One. 2013 Jan 30;8(1):e55219. doi: 10.1371/journal.pone.0055219 (PMC3559389; doi:10.1371/journal.pone.0055219)
Supplement: Table S1 — Clinical isolates in this study along with their respective origins and properties. (DOC) [file pone.0055219.s001.doc]

**Supporting information**

**Table S1. Clinical isolates in this study along with their respective origins and properties**

***: D, dead; C, cure**

****: +, detected; −, not detected**

**rRNA, ribosomal ribonucleic acid; CPS, capsular polysaccharide.**
